# Herbivory can increase plant fitness via reduced interspecific competition—evidence from models and mesocosms

**DOI:** 10.1098/rspb.2024.1149

**Published:** 2025-01-22

**Authors:** Laura Böttner, Fabio Dudenhausen, Sara Nouere, Antonino Malacrinò, Martin Schäfer, Joris M. Koene, Meret Huber, Shuqing Xu

**Affiliations:** ^1^ Institute of Plant Biology and Biotechnology, University of Münster, Münster 48143, Germany; ^2^ Institute for Evolution and Biodiversity, University of Münster, Münster 48149, Germany; ^3^ Institute of Organismic and Molecular Evolution, Johannes Gutenberg University Mainz, Mainz 55128, Germany; ^4^ Department of Agriculture, Università degli Studi Mediterranea di Reggio Calabria, Reggio Calabria 89122, Italy; ^5^ Amsterdam Institute for Life and Environment, Section Ecology & Evolution, Vrije Universiteit, Amsterdam 1081 HV, The Netherlands; ^6^ Institute for Quantitative and Computational Biosciences, Johannes Gutenberg University Mainz, Mainz 55128, Germany

**Keywords:** duckweed (*Spirodela polyrhiza*), algae, interspecific competition, intraspecific competition, plant–herbivore interactions, plant fitness

## Abstract

Herbivores are generally considered to reduce plant fitness. However, as in natural communities they often feed on several competing plant species, herbivores can also increase plant fitness by reducing interspecific competition among plants. In this study, we developed a testable model to predict plant fitness in the presence of an interspecific competitor and a herbivore that feeds on both plant species. Our model allows prediction of the herbivore and competitor densities at which the focal species will benefit from herbivory. This can be estimated by quantifying the effects of the herbivore on the fitness of the focal plant and on its competitor, and by estimating the levels of intra- and interspecific competition in a pairwise fashion, respectively. We subsequently validated the model in indoor microcosms using three interacting species: an aquatic macrophyte (the giant duckweed *Spirodela polyrhiza*), its native competitors (green algae) and its native herbivore (the pond snail *Lymnaea stagnalis*). Additional outdoor mesocosm experiments supported our model under natural conditions. Together, this study provides a conceptual framework to understand how herbivores shape plant fitness in a community context.

## 1. Introduction

Plants are the backbone of most ecological interactions, and plant–herbivore interactions shape most of the planet’s ecosystems [1–3]. Herbivores are usually assumed to reduce plant fitness as herbivores consume plant material and thereby reduce growth and eventually reproduction (direct fitness costs). However, species do not occur in isolation but in a community, and herbivores often feed not only on a single but on multiple competing plant species. Thus, if a herbivore reduces the growth of a competitor, the herbivore may increase the fitness of the focal species, even when the focal species is grazed upon (indirect fitness benefits through reduced competition). For example, molluscs may increase the growth of grasses (*Festuca rubra*) by decreasing the abundance of herbs [4]. Similarly, insect herbivores feeding on goldenrod populations indirectly led to a long-term increase in understorey forb abundance by increasing light availability [5]. These studies illustrate that herbivory can have indirect positive fitness effects on the focal species by reducing interspecific competition, which may outweigh the direct negative effects of being eaten by the herbivore. However, we currently lack a theoretical framework to quantitatively predict the effects of herbivory on focal plants when they are also competing with other species (e.g. considering the initial densities of both the focal and competing species).

Quantitatively predicting the net fitness impact of herbivores on plant groups and quantifying competition outcomes can be crucial for maintaining healthy aquatic environments. Herbivory can mitigate the effects of nutrient enrichment on plant diversity by controlling dominant species, which has direct implications for managing eutrophication and algal blooms exacerbated by human activity [6,7]. In terrestrial systems, changes to nutrient cycles and herbivore communities affect global biodiversity where herbivory may counteract plant species losses due to eutrophication by enhancing ground-level light availability in grasslands [8,9].

Predicting the net fitness effect of herbivores can be achieved by modifying and combining existing models of predator–prey interactions and two-species competition models [10–16]. Combining mechanistic modelling with model-guided experiments can help to understand microcosm functioning of competing species, e.g. under abiotic stress [11]. Importantly, empirical studies should assess the consequences of herbivory and competition not only on plant phenotypes and growth, but also on plant fitness, that is, their contribution to the gene pool of the next generation [17]. However, this is challenging because many plants have long life cycles. Long-term herbivory exclusion experiments can overcome this limitation and thereby reveal the consequences of herbivory on plant abundance, community structure [18,19], diversity [4] and performance of alien plants under invasion [20]. However, in these long-term outdoor exclusion experiments, herbivores and competing plants continuously affect each other’s density, which prevents teasing apart, and therefore, quantifying the direct and indirect effects of herbivory. To test theoretical predictions when positive indirect effects outweigh the negative direct effects of herbivores, we need an experimental system in which we can manipulate the densities of both plant species.

One of the few experimental systems in which the densities of the plant, its herbivore and its competitor can be easily manipulated is the duckweed–snail–algae system. Within freshwater ecosystems, the balance between free-floating plants (such as duckweeds) and submerged species (e.g. macrophytes, macroalgae, free-swimming microalgae and photosynthetically active cyanobacteria) plays a crucial role in shaping the habitat structure and providing niches for various organisms, including fish, invertebrates and microorganisms [21,22]. These plant groups intra- and interspecifically compete for light and nutrients and can be attacked by the same herbivore [23–25]. Duckweeds are free-floating, aquatic plants of small body size and simple morphology: the green vegetative tissue forms a flat, thallus-like structure, the so-called frond. Like most duckweed species, the giant duckweed, *Spirodela polyrhiza* (L.) Schleid, reproduces almost exclusively asexually by budding every 2–3 days under optimal conditions indoors [26,27] and every 3–7 days outdoors. Outdoors, the plant is frequently attacked by one of its major native herbivores, the holarctic common freshwater snail *Lymnaea stagnalis*. This snail feeds not only on *S. polyrhiza* but also on green algae, such as the microalgae *Chlamydomonas* spp., which compete with *S. polyrhiza* for light and nutrients [24,28]. As the abundances of *S. polyrhiza*, its herbivore *L. stagnalis* and its competitor *Chlamydomonas* spp. can be quantified and manipulated across multiple generations [29], these interacting species are ideal for testing the direct and indirect effects of herbivores on plant fitness.

Here, we first developed a theoretical model to understand the interaction among *S. polyrhiza*, its herbivore *L. stagnalis*, and its competitor *Chlamydomonas reinhardtii*. Second, we used a reductionist setup for indoor model-guided assays to feed our theoretical model with data. Third, we performed indoor microcosm experiments as a proof of concept to validate our model, testing whether a herbivore promotes *S. polyrhiza* fitness in the presence of a competitor, and whether this effects depends on the abundance of the competing species. Fourth, we tested whether our findings hold true under outdoor conditions by quantifying the algal biomass and characterizing the algal community using amplicon metagenomics. Our findings highlight the importance of considering the real-world complexity when assessing the effects of herbivores on plant fitness.

## 2. Material and methods

### (a) Model development and estimation of model parameters using laboratory microcosms

First, we built a generic model that incorporates both plant–herbivore interactions and plant–plant competition based on existing species-competition models [10–16].

For simplicity, we focused on two competing species that can be attacked by the same herbivore. The population growth of the two plant species, which is affected by competition (intra- and interspecies) and herbivory, can be expressed as

(2.1) $$\frac{\text{d}N_{1}}{\text{d}t}=r_{1}N_{1}\left(1-a_{11}N_{1}-\;a_{12}N_{2}-\beta_{1}N_{\text{h}}\right)$$

and

(2.2) $$\frac{\text{d}N_{2}}{\text{d}t}=r_{2}N_{2}\left(1-a_{22}N_{2}-\;a_{21}N_{1}-\beta_{2}N_{\text{h}}\right),$$

where $r_{1}$ and $r_{2}$ are the intrinsic growth rates of the two species. $N_{1}$ and $N_{2}$ are the numbers of individuals from the two species at a given time. $a_{11}$ and $a_{22}$ refer to intraspecific competition. $a_{12}$ and $a_{21}$ are interspecific competition coefficients describing the effects of the species associated with the second number in the subscript on the species associated with the first number in the subscript. $\beta_{1}$ and $\beta_{2}$ refer to herbivore consumption rates of species 1 and 2, respectively. $N_{\text{h}}$ refers to herbivore abundance. For simplicity, we assumed a constant herbivore population size, mirroring natural scenarios where the herbivore (e.g. large grazers) life cycle often exceeds that of the plant life cycle, as is relevant to our model system.

To test our theoretical model with experimental data, we used model-guided laboratory two-species assays, which allowed us to estimate the model coefficients using R [30]. As model species, we used *S. polyrhiza* (genotype 9500, originating from Germany), *C. reinhardtii* (initial culture originating from a natural pond in Münster, Germany) and simultaneously hermaphroditic pond snail *L. stagnalis* (initial population originating from a long-standing laboratory culture from the Vrije Universiteit Amsterdam, The Netherlands [31]). Precultivation and indoor assays were carried out in a growth chamber at 26°C with a light intensity of 135 µmol m^−2^ s^−1^ under long-day conditions (16 h : 8 h). All species were first cultivated in individually optimized media [32] (electronic supplementary material, table S1) and then preadapted to common experimental conditions (electronic supplementary material, text S1 and table S2). The snails were starved for 24 h under experimental conditions prior to the assays. For indoor assays, we used transparent plastic beakers (diameter 11 cm, polypropylene) with 150 ml of Mix-Medium (electronic supplementary material, table S2). The beakers were covered with dark foil to avoid the incidence of light, and beakers were covered with transparent and perforated plastic lids to ensure gas exchange but to prevent evaporation and escape of herbivores.

To quantify *C. reinhardtii*, we generated a reference curve using a Thoma cell counting chamber and measured the corresponding optical density (OD) at 750 nm with a plate reader (Tecan Infinite 200 with the software MagellanPro v. 7.3 2019) [29], with pure Mix-Medium as a blank, covering a *C. reinhardtii* concentration range from 0 to 22 [10^6^ cells ml^−1^] (electronic supplementary material, figure S1).

To estimate the intra- (*α*_11_) and interspecies (*α*_12_) coefficients of *S. polyrhiza* of our theoretical model, we quantified fitness, measured in terms of the number of individuals (expressed as *per capita* growth) of *S. polyrhiza* when competing against varying densities of itself, as well as when grown in the presence of a constant concentration of *C. reinhardtii* as a competitor. We grew duckweed with a starting population size of 5, 50, 100, 300, 600 or 1000 fronds in triplicate in the absence and presence of a fixed algae concentration of 1.33 [10^6^ cells ml^−1^]. We reset the algae concentration to 1.33 [10^6^ cells ml^−1^] daily for seven consecutive days, using centrifugation. To maintain the same medium with respective nutrient level changes throughout the course of the experiment, we reused the supernatant medium of each individual experimental beaker and added newly cultivated algae cells (daily pre-adapted to Mix-Medium) at the respective concentrations. To quantify *S. polyrhiza* on day 7 of the experiment*,* we counted the number of live fronds, i.e. green fronds with intact reproductive pouches. All the visible small daughter fronds were counted as individuals. *Per capita* growth per day is expressed using the formula $\ln(N_{t0}+\Delta_{t}/N_{t0})/\Delta_{t}$, where $N_{t0}$ refers to the initial frond number, $N_{t0}+\Delta_{t}$ refers to frond number after 7 days, and *$\Delta_{t}$* is 7 days.

To estimate the coefficients *α*_22_ and *α*_21_, describing the intra- and interspecific coefficients of *C. reinhardtii*, we grew algae at defined starting concentrations (0.0095, 0.59, 0.13, 1.64, 3.91, 7.66, 9.24 [10^6^ cells ml^−1^] in the absence (*n* = 4 per gradient step) and presence (*n* = 4 per gradient step) of 100 fronds. To keep the number of fronds constant across 7 days, we manually reduced duckweed to 100 individuals on days 2 and 5 of the experiment. *Per capita* growth was calculated analogously to the *per capita* growth of *S. polyrhiza*. Here, we assume a linear relationship for the interspecific competition.

To determine the individual feeding rate of *S. polyrhiza* (*β*_1_) by a snail within 24 h, we subjected 100 fronds to one (*n* = 6) or no snail as a control (*n* = 4). We harvested all remaining fronds after 24 h. Similarly, to determine the individual feeding rate of *C. reinhardtii* (*β*_2_) by a snail within 24 h, we started with a concentration of 12.6 [10^6^ cells ml^−1^] and one snail (*n* = 8) or no snail as control (*n* = 4). We again measured the OD after 24 h to determine the remaining algae concentration. For all consumption rates, we assumed a constant feeding rate within 24 h. An overview of model parameters and units can be found in electronic supplementary material, table S3.

The data from the experiments described above allowed us to estimate the competition coefficients (*α*_11_, *α*_22_, *α*_12,_ *α*_21_) and consumption coefficients (*β*_1_, *β*_2_) based on our proposed model equations using R [30] (see electronic supplementary material, R Markdown script). Data were visualized using *ggplot2* [33].

The 95% confidence intervals of each parameter were estimated using a bootstrapping approach with 500 repetitions. Based on the extracted coefficients, we were able to test the predicted scenarios from electronic supplementary material, figure S2 to determine the scenario that best describes the duckweed–algae–snail interaction.

### (b) Testing our theoretical model within laboratory microcosms

We tested within-laboratory three-species microcosms, under which starting conditions *S. polyrhiza* benefits from the presence of a snail herbivore when competing with *C. reinhardtii*. We included a treatment with a high number of duckweed fronds competing against a low concentration of algae, and *vice versa*, as well as two treatments with intermediate densities. We grew these combinations with either no snails, one snail or two snails. We excluded replicates in which the populations collapsed, or snails died within 14 days from the analysis, resulting in a dataset of *n* = 3–8 per treatment. The details can be found in electronic supplementary material, text S2. On day 14 of the experiment, we counted the number of live fronds, measured the OD of the algae and calculated the *per capita* growth of both competing species. Data were analysed using Wilcoxon tests to compare *per capita* growth between the herbivory and control treatments.

### (c) Experimental validations in the field

To test our model in a natural setting, we set up an outdoor experiment in 2019 in Münster, Germany (51°57′54.0′′ N, 7°36′22.4′′ E), in which *S. polyrhiza* was exposed to a natural community of green algae in the presence and absence of snail herbivory. Eight experimental ponds (60 × 80 × 32 cm; AuerPackaging, Amerang, Germany) were buried up to their rims in the soil and filled on 26 June 2019 with 120 l of tap water, 6 l of commercial pond soil (approx. 100 mg l^−1^ each of organic N, P_2_O_5_ and K_2_O, *ca* 160 mg l^−1^ Mg, pH (CaCl_2_) = 4.2, salts 1.0 g l^−1^; Floragard, Oldenburg, Germany), and 40 ml of commercial liquid fertilizer (organic NPK 3.1 + 0.5 + 4.1; COMPO BIO, Compo, Münster, Germany), based on pretests allowing sufficient growth similar to natural ponds. To allow the establishment of a natural (phyto-)plankton community, ponds were inoculated with filtered water (via a coffee filter) from three natural ponds located within and around Münster municipality, where duckweeds (*S. polyrhiza* and *Lemna* spp.) naturally occur (electronic supplementary material, table S4). The experimental ponds were covered with a stainless-steel mesh (mesh opening 0.63 mm, wire diameter 0.224 mm; Haver & Boeker, Oelde, Germany) to avoid debris and small animals entering the ponds. The ponds were divided into two compartments by using the same metal net. Each compartment received 1000 *S*. *polyrhiza* fronds that had been pre-cultivated indoors (electronic supplementary material, text S1). The plants were shaded with a shading net during the first week to facilitate plant establishment. Then, on 15 July, the experiment began with eight snails (shell size 14–32 mm), which were added to one of the compartments of each pond; the other compartment served as a control. As the two compartments were separated with a fine metal net, water and dissolved nutrients, but not herbivores, could be exchanged between compartments. To compensate for the water loss due to evaporation, the ponds were refilled with tap water once a week. In week 6, aphids invaded three ponds and extinguished duckweed populations. To avoid further contamination, we removed infested ponds from the field and excluded all their data from our analysis. Therefore, all the data presented correspond to *n* = 5 per treatment until week nine, which was the end of the experiment.

#### (i) Focal species sampling and fitness measurements in the field

To track the growth of the duckweed populations, we took pictures of the populations weekly and estimated the surface area covered by plants over time using the ImageJ software [34]. Normalized fitness change was calculated for each timepoint by using the percentage coverage rates with the formula: $\left(SA_{herb}-SA_{ctr}\right)/Max\left(SA_{herb},\;SA_{ctr}\right)$, where $SA_{herb}$ and $SA_{ctr}$ refer to surface area (SA) in the presence (herbivory) and absence (control) of snails. We further tested whether herbivory altered the biomass of fronds by sampling 20 randomly selected fronds from each treatment zone and pond at weeks 0, 2, 3, 4, 5, 7, 8 and 9. Subsequently, we briefly dried fronds with paper towels and measured their fresh biomass.

The effect of herbivory on plant fitness measured via surface area coverage and biomass was tested by fitting a linear mixed effect model, using coverage or biomass within each treatment zone as variable and specifying *treatment* (herbivory/control) as fixed factor, and *pond* and *timepoint* as random factors, using the formula: fitness = treatment + (1|timepoint) + (1|pond) using the lme4 package [35].

#### (ii) Water nutrient measurements in the field

To test whether water exchange through the net maintained equal nutrient levels between the herbivore and control compartments, we analysed the nutrients in each treatment zone per pond via ion-chromatography (792 basic IC, 732 detector, Metrohm, Herisau, Switzerland). Samples were collected in weeks 0, 3, 5, 7 and 9. We sampled 10 ml per treatment zone for each anion and cation analysis. Water was collected from two locations within each zone at a depth of 10 cm below the water surface. Anion (Cl^−^, $NO_{2}^{-}$, $NO_{3}^{-}$, $PO_{4}^{3-}$, $SO_{4}^{2-}$) samples were measured right away, while pH of cation samples (for Na^+^, $NH_{4}^{+}$, K^+^, Ca^2+^, Mg^2+^) was adjusted to pH 3 using 25% HNO_3_ before storage at −20°C until analysis.

The effect of herbivory on water nutrients was tested by fitting a linear mixed effects model specifying *treatment* (herbivory/control) as fixed factor, and *pond* and *timepoint$NO_{3}^{-}$* as random effects, using the formula: nutrient = treatment + (1|timepoint) + (1|pond). In addition, we tested the effects of overall nutrient content through permutational multivariate analysis of variance (PERMANOVA) with 999 permutations, stratified over *pond* and *sampling timepoint*.

The water temperature was measured just below the water surface, within the zone of the duckweed, using a data logger (Hobo Pendant MXTemp, Onset Computer Corporation*,* MA, USA). Data for air temperature were provided by a weather station about 1 km away from the site.

#### (iii) Competitor sampling and fitness measurements in the field

We also collected algal samples from the field. We first identified the emerging algal species based on their morphology. We characterized free-swimming microalgae emerging within the first half of the season, as well as sediment-rooted macroalgae that emerged during the second half of the season. To test whether herbivory alters the biomass of sediment-rooted macroalgae, we harvested all sediment-rooted macroalgal materials at the end of the season. The harvested macroalgae material was washed several times in tap water, frozen at −20°C, and freeze-dried. Dry weight was determined and analysed using a linear mixed effect model with *treatment* (herbivory/control) as a fixed factor and *pond* as a random factor, using the formula: dry weight = treatment + (1|pond).

To test whether macro- and micro-algae and associated (photosynthetically active cyano-)bacterial communities differ under herbivory and control conditions outdoors, we used an amplicon metagenomic approach. We selected two types of samples as follows. First, we sampled 30 fronds of each treatment and control pond (*n* = 5) at weeks 0, 4 and 7 to characterize the free-swimming microalgae (18S) and (cyano-)bacteria (16S) communities that were attached to or growing as endophytes of *S. polyrhiza* fronds. Upon harvest, the fronds were washed with ultrapure water and frozen at −20°C. Second, we harvested macroalgal material with adherent microalgae at the end of the season, as described above, to characterize the (macro-)algal community (18S) of each treatment and control pond (*n* = 4 or 5, as one herbivore compartment did not contain any macroalgae).

Freeze-dried fronds were ground with steel balls inside a tissue lyser (1.5 min at 30 Hz). DNA was extracted using a phenol–chloroform protocol. Samples were then shipped to Novogene (Beijing) for amplicon metagenomic library preparation and sequenced on an Illumina NovaSeq 6000 S4 250PE flow cell. (Cyano-)bacterial communities were assessed by amplifying the V4 region of the 16S rRNA (primers 515F and 806R), whereas the eukaryotic community (including algae) was determined by amplifying the V4 region of the 18S rRNA (primers 528F and 706R).

Macroalgal material with adherent microalgae was freeze-dried, weighed and ground using a mortar and pestle. DNA was extracted from approximately 30 mg of freeze-dried ground tissue using the Plant II Mini Kit (Machery-Nagel, Düren, Germany) according to the manufacturer’s instructions with the following adjustments: 400 µl P1 (CTAB-based), lysis for 1 h at 65°C and elution for 20 min at 65°C. The samples were then subjected to StarSeq (Mainz, Germany) for library preparation (18S, primers 528F and 706R) and sequencing using Illumina MiSeq (300PE).

Raw data were processed using *TrimGalore* v. 0.6.7 to remove Illumina adaptors and discard low-quality reads. Paired-end reads were processed using the *DADA2* v. 1.22 [36] pipeline implemented in R to remove low-quality data, identify amplicon sequence variants (ASVs) and remove 10 chimeras. Taxonomy was assigned using the *SILVA* v. 138 database [37] for 16S data or the *PR2* v. 4.14 database [38] for 18S data. Data were analysed using R v. 4.1.2, with the packages *phyloseq* v. 1.38 [39], *vegan* v. 2.6 [40], *DESeq2* 1.34 [41] and *lme4* 1.1.33 [35]. Analysis of 18S was restricted to the phylum ‘Archaeplastida’, which includes green algae. Before downstream analyses, contaminants were removed using the *decontam* v. 1.14 [42] and data from non-template control libraries. We also removed reads identified as ‘chloroplast’ or ‘mitochondria’ and singletons.

Community composition distances between sample pairs were calculated using an unweighted UniFrac matrix using *phyloseq* v. 1.38 [39], which measures the phylogenetic distance between taxa or genera by calculating the fraction of branch length in a phylogenetic tree that is unique to either one treatment or the other, but not shared by both [43]. These distances were then visualized using a non-metric multidimensional scaling (NMDS) procedure. Differences between sample groups were inferred through PERMANOVA (999 permutations), specifying *treatment* (herbivory/control) as a fixed factor, and using the factor *pond* to stratify permutations. Differences in microbial diversity between groups were assessed by calculating the Shannon diversity index and by fitting a linear mixed effects model using the formula Shannon_index = treatment * timepoint + (1|pond). ASVs that were differentially abundant between treatments (herbivory/control) were identified using *DESeq2* with false discovery rate (FDR)-corrected *p* < 0.05.

## 3. Results

### (a) A model for plant fitness consequences under herbivory in the presence of a competitor

**Figure 1.**
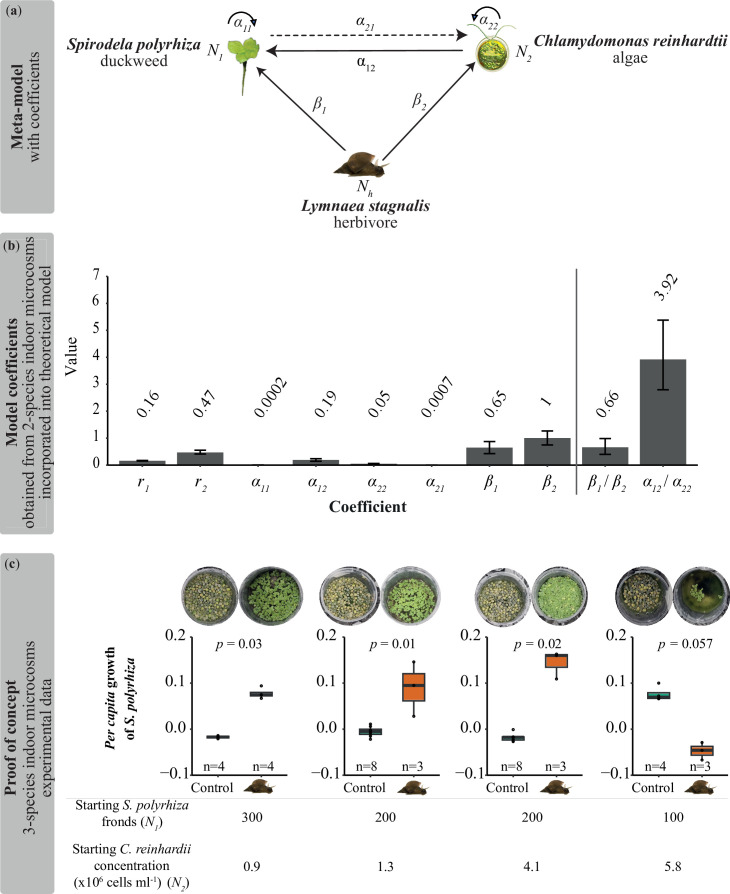
Modelling approach and experimental proof of concept to test under which starting conditions duckweed benefits from herbivory when competing with algae. (a) Meta-model representing the causal relationships among our focal plant species *Spirodela polyrhiza*, the green microalga *Chlamydomonas reinhardtii* and the herbivorous snail *Lymnaea stagnalis*. Both competing species underlie intraspecific (*α*_11_, *α*_22_) and interspecific (*α*_12_, *α*_21_) competition and a fixed abundance of the herbivore ($N_{h}$) has individual consumption rates of each organism (*β*_1_, *β*_2_). (b) Estimated coefficients based on two-species laboratory assays (mean and 95% confidence interval). Numbers above bars give mean values. Based on our model, species 1 benefits under herbivory if $\beta_{1}/\beta_{2}<a_{12}/a_{22}$. (c) Three-species indoor-microcosm data as proof of concept. *Spirodela polyrhiza* benefitted under herbivory in the presence of *C. reinhardtii* as competitor. The effects are mostly consistent among different starting population sizes of the two competing species. One exception is when the ratio of *C. reinhardtii* and *S. polyrhiza* is very high, which could be due to changes in the feeding behaviour that increased the ratio of $\beta_{1}/\beta_{2}$. *p*-values refer to Wilcoxon tests, *n* = 3–8. The picture of *C. reinhardtii* was created with Biorender.

We proposed a testable theoretical model describing the interaction of a focal species 1, its competing species 2 and an herbivore feeding on both species (meta-model, figure 1*a*). The model aimed to predict under which starting abundances of each species our focal species will benefit from herbivory by reducing interspecific competition. Based on equations (2.1) and (2.2), we derived the isoclines of two species, and the lines that represent the zero-growth rate of each species can be expressed as:

(3.1) $$N_{1}=\left(1-\;a_{12}N_{2}-\beta_{1}N_{\text{h}}\right)/a_{11}$$

and

(3.2) $$N_{2}=\left(1-\;a_{21}N_{1}-\beta_{2}N_{\text{h}}\right)/a_{22}.$$

A graphic representation of the isoclines is shown in electronic supplementary material, figure S2. To understand the conditions under which herbivory promotes species growth, we calculated the invasion growth rate (IGR) of the focal species (i.e. species 1) with herbivory (IGR_w_) and without herbivory (IGR_wo_), which can be expressed as:

(3.3) $${\text{IGR}}_{\text{w}}=r_{1}\left(1-\;a_{12}N_{2}^{\text{w}}-\beta_{1}N_{\text{h}}\right),$$

(3.4) $${\text{IGR}}_{\text{wo}}=r_{1}\left(1-\;a_{12}N_{2}^{wo}\right),$$

where $N_{2}^{\text{w}}$ and $N_{2}^{wo}$ refer to the population size of species 2 (in equilibrium), when species 1 is absent, with and without herbivore. Based on equations 3.1 and 3.2, $N_{2}^{\text{w}}$ and $N_{2}^{wo}$ can be expressed as:

(3.5) $$N_{2}^{\text{w}}=\left(1-\beta_{2}N_{\text{h}}\right)/a_{22},$$

(3.6) $$N_{2}^{wo}=1/a_{22},$$

For conditions where herbivory promotes the growth of the focal species (i.e. species 1), we must have

(3.7) $$IGR_{w}>IGR_{wo}.$$

Solving equations (3.3)–(3.7) gives $\beta_{1}/\beta_{2}\;<\;\;a_{12}/\;a_{22}$. This can be interpreted as the ratio of herbivory effects on the two species being less than the ratio of competitiveness of species 2 against species 1 and intraspecific competition of 2. Interestingly, the model predicts that herbivory effects on the focal species are independent of the population densities of both species. Similar conditions also apply to species 2. An overview of how prior conditions affect predicted coexistence and herbivory effects can be found in electronic supplementary material, table S5.

### (b) Testing model predictions using laboratory microcosms

Feeding our theoretical model with experimental data of model-guided two-species assays allowed us to estimate both intra- and interspecific competition coefficients, as well as individual consumption rates (figure 1*b*). Here, we first grew duckweed and algae either separately or together in beakers. Overall, the interspecific competition (in the absence of herbivory) between algae and duckweed was asymmetric (electronic supplementary material, figure S3a,b). Although algae strongly reduced duckweed growth, duckweed did not significantly affect algal growth. Second, quantifying consumption rates (in the absence of a competitor) of snails on duckweed and on algae suggested that snail herbivory reduced the growth of both algae and duckweed (electronic supplementary material, figure S3c,d); however, the effect of herbivory on algae was greater than that on duckweed.

Based on the estimated parameters and coefficients (figure 1*b*), our model predicted that duckweed and algae would coexist in the absence of snails, but the duckweed population would remain small (electronic supplementary material, figure S2). However, in the presence of a snail, duckweed will perform better and outcompete algae (because $\beta_{1}/\beta_{2}\;<\;\;a_{12}/\;a_{22}$; electronic supplementary material, figure S2). Our model also predicted that if the number of snails was too high (greater than two in our experimental laboratory setup using beakers), both the duckweed and *C. reinhardtii* populations would collapse.

To test these predictions, we grew duckweed and algae in the presence and absence of snails for 14 days in indoor three-species microcosms using beakers. As predicted, two snails often extinguished the populations. Therefore, we focused on comparisons between one and no snails. In three out of four scenarios, where the algae density was relatively low or intermediate high, snails indeed increased the duckweed growth rate (*p* ≤ 0.02*,* Wilcoxon test, *n* = 3–8, figure 1*c*). This is highly consistent with the model prediction.

However, when the starting algae density was very high (5.8 [10^6^ cells ml^−1^]) and the starting duckweed population size was low (100 fronds), snail herbivory also led to the collapse of the duckweed population (figure 1*c*), whereas the duckweed population increased in the absence of snail herbivory. Therefore, in such conditions, snail herbivory reduced duckweed fitness.

Our model also predicts that snails always reduce algal population size ($\beta_{2}/\beta_{1}\;>\;\;a_{21}/\;a_{11}$), as $\beta_{2}$ is greater than 0 and $a_{21}$ equals 0 (statistically), independently of duckweed population density. In our experiments, the algal populations grew significantly worse in the presence of snails under nearly all tested conditions (*p* ≤ 0.03, Wilcoxon test, *n* = 3–8; electronic supplementary material, figure S4).

### (c) Quantifying herbivory effects in outdoor mesocosms

**Figure 2.**
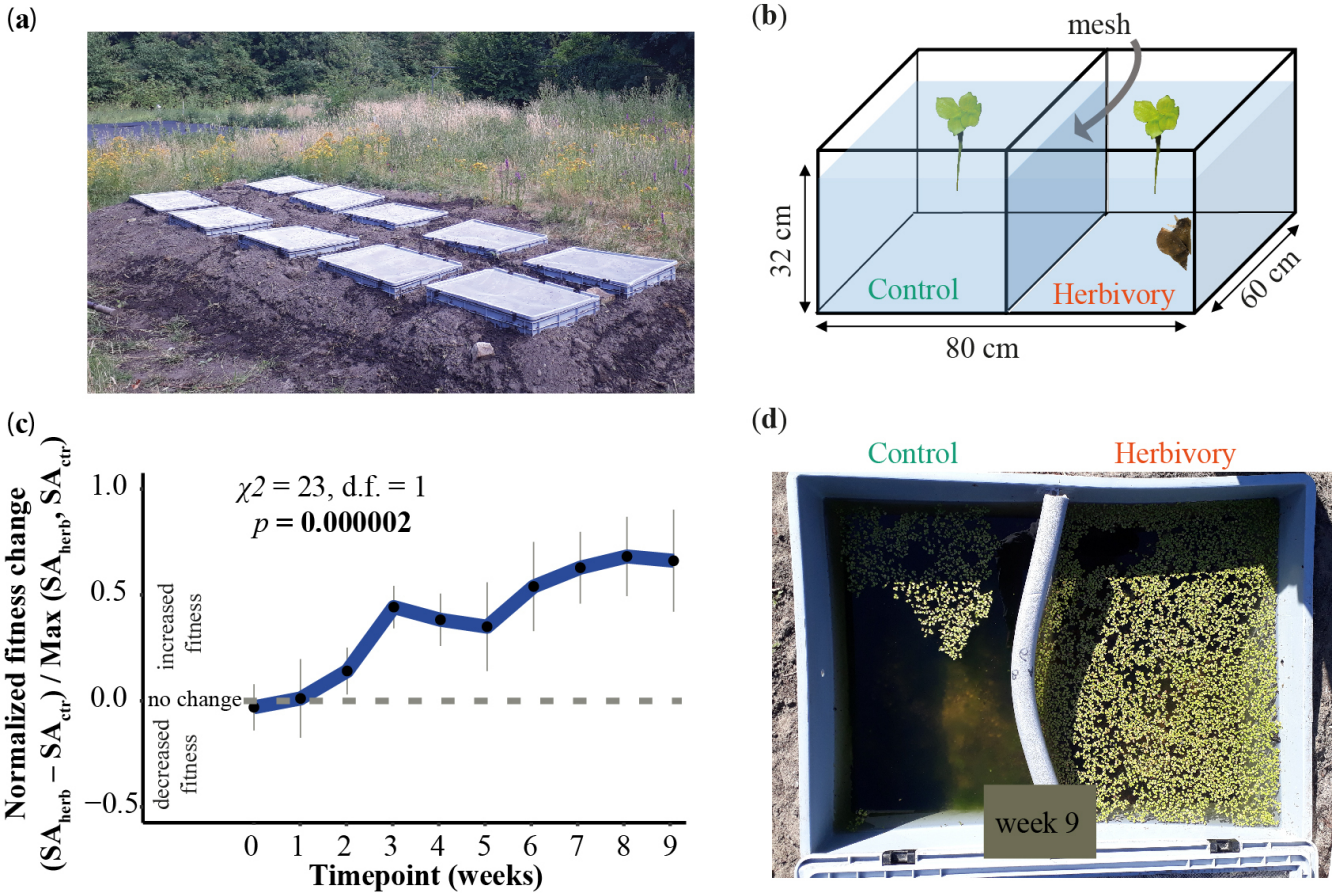
Herbivory treatment increased relative fitness of *Spirodela polyrhiza* in outdoor mesocosms. (a) Field site with eight replicated ponds covered with mesh. (b) Schematic representation of each pond. Treatment compartments with presence and absence of the snail herbivore *Lymnaea stagnalis* were separated by a mesh, preventing snails from entering the control compartment but allowing water exchange. (c) Presence of *L. stagnalis* increased the fitness of *S. polyrhiza* outdoors over a course of nine weeks during one summer season, representing about 15–20 asexual generations of plants (*n* = 4 or 5, linear mixed effect model, SA = surface area, herb = herbivory, ctr = control). Values above 0 indicate herbivory increased duckweed fitness. The *y*-axis refers to normalized fitness and the *x*-axis refers to time. (d) Representative plant populations in week 9.

We tested whether snail herbivory can increase duckweed growth via reduced competition under outdoor conditions by growing replicated monoclonal duckweed populations and their native algal community in the presence and absence of snails across one growth season in outdoor ponds (figure 2*a*,*b*). Three weeks after the start of the experiments, the snail-infested duckweed populations covered an average of a 57% larger surface than control populations, and this difference even grew towards the

end of the experiment (*p* = 0.000002, *χ*^2^ = 22.5, linear mixed effect model; figure 2*c*,*d*). Increased duckweed coverage occurred despite the duckweed's being attacked by the snails. The increased duckweed surface coverage cannot be explained by an increased biomass per frond, as biomass per frond was reduced rather than increased under herbivory at week 4 (*p* = 0.002, linear mixed effect model; electronic supplementary material, figure S5), with diminishing effects in the following week. Increased duckweed growth under snail herbivory was likely not due to altered nutrient levels, as nutrient levels did not differ significantly between the adjacent snail and herbivore compartments (electronic supplementary material, table S6).

**Figure 3.**
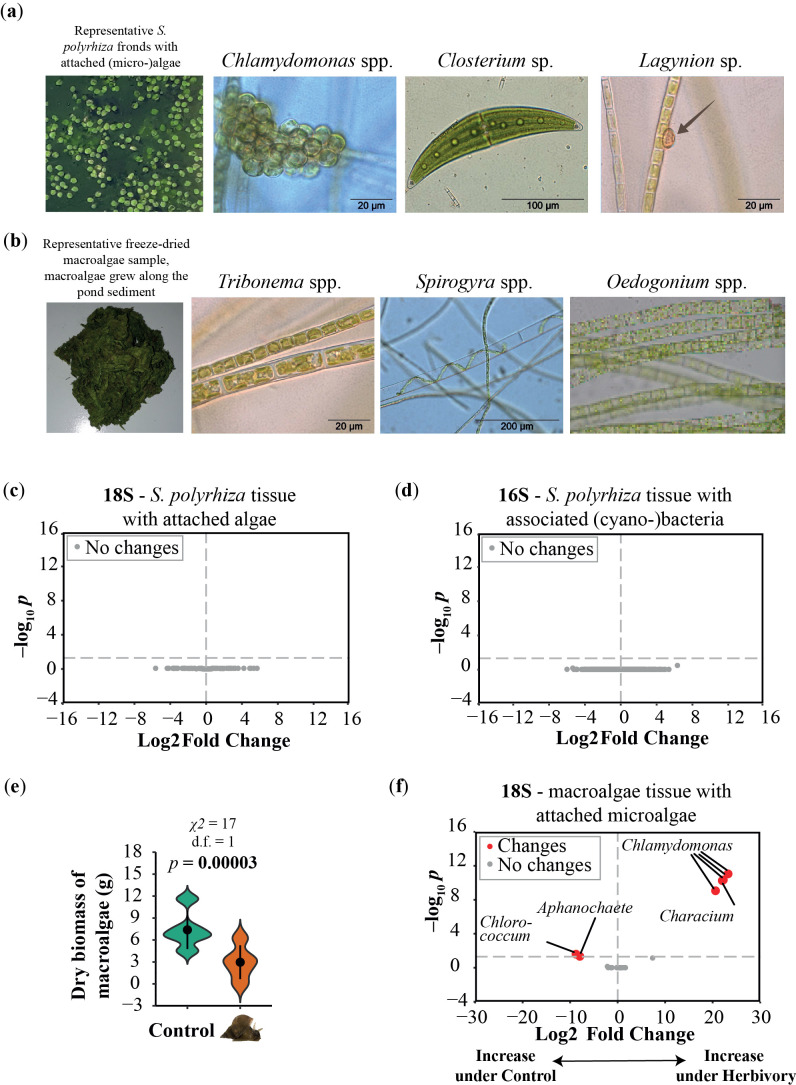
Algal community lived together with *Spirodela polyrhiza* in outdoor mesocosms. Herbivory treatment reduced the abundance of macroalgae biomass and partly affected the abundance of microalgae genera associated with macroalgae samples in outdoor ponds. (a) In early summer, the abundance of microalgae was high. Representative pictures of dominant microalgae species, later confirmed via amplicon metagenomic approach. (b) During the second half of the season, macroalgae started growing. Representative pictures of dominant macroalgae species harvested at the end of the season, later confirmed via amplicon metagenomic approach. (c) Presence of snails did not affect the abundance of different genera of algae (18S) and (d) (cyano-)bacteria (16S) associated with *S. polyrhiza* samples (grey dots). (e) Presence of snails significantly reduced macroalgae abundance (linear mixed effect model, *n* = 5). (f) Herbivory affected the relative abundance of 18S genus hits associated with macroalgae samples. Significantly higher abundance of genera (red dots) in either control treatment (log_2_ fold changes <0) or herbivory treatment (log_2_ fold changes >0). Non-significant changes in abundance are depicted in grey. Microscopy pictures were taken by Antje Gutowski.

Shortly after setting up the experiment, we observed rapid growth of microalgae, concurrent with an increase in water and air temperature (electronic supplementary material, figure S6), with a peak at week 4. Based on morphology, we identified *Chlamydomonas* spp. as the dominant microalgal species, accompanied by *Closterium* spp. and *Lagynion* spp. (figure 3*a*). Amplicon metagenomics using the 18S rRNA gene marker on duckweed samples with adherent microalgae revealed that the dominant microalgae were Chlamydomonadales (electronic supplementary material, figure S7). Herbivory treatment did not affect microalgal and cyanobacterial community structure (electronic supplementary material, figure S8a,b), neither the microbial diversity associated with *S. polyrhiza* fronds (*p* > 0.2, PERMANOVA; electronic supplementary material, figure S7 and table S7) nor the relative abundance of different algal genera (figure 3*c*,*d*). However, our method was unable to estimate the absolute quantity of microalgae.

In the second half of the experimental period (weeks 4−9), thick clumps of filamentous macroalgae started to grow along the bottom of the pond (figure 3*b*) and eventually started to attach to the duckweed roots. At the end of the growing season, these macroalgae were significantly less abundant in the presence than in the absence of the snail herbivore (*p* = 0.00003, linear mixed-effect model; figure 3*e*). An additional feeding experiment also showed that the snails could directly feed on the macroalgae collected from the ponds (electronic supplementary material, figure S9). Based on morphology, we identified *Tribonema* spp., *Oedogonium* spp. and *Spirogyra* spp. (figure 3*b*) as the dominant macroalgal species, which was further confirmed by 18S amplicon sequencing (electronic supplementary material, figure S10). Again, based on 18S rRNA gene marker, Chlamydomonadales were the dominant microalgae associated with macroalgae samples. Shannon diversity of macroalgae with adherent microalgae was significantly higher under herbivory (*p* = 0.03, linear mixed effect model; electronic supplementary material, figure S10), whereas community structure was unaffected by herbivory (electronic supplementary material, figure S8c). Furthermore, herbivory affected the abundance of different genera associated with macroalgae samples, for example, a higher relative abundance under herbivory for *Chlamydomonas* (figure 3*f*).

Together, these results are consistent with our model prediction that snails increase duckweed fitness by reducing the growth of their competitors, here the macroalgae, under outdoor conditions.

## 4. Discussion

Herbivory and competition are two major factors that can influence plant fitness. Although these two factors are often studied separately, they are intertwined, as herbivores can alter the competitive landscape when feeding on multiple species. Here, we provide parallel evidence that herbivory can increase plant fitness via reduced interspecific competition. First, we developed a theoretical model to understand the fitness consequences of competing (plant) species under herbivory. We found that herbivory effects on the fitness of the focal species are determined by the ratio of inter- and intra-competition ($a_{12}/a_{22}$) relative to the consumption rates of focal and competing species ($\beta_{1}/\beta_{2}$). Second, we tested and validated our model predictions using three aquatic species (including microalgae) growing together in an indoor microcosm. Third, the outdoor mesocosms confirmed that herbivory can increase plant fitness by reducing competing species (here, macroalgae) under natural conditions.

We developed our model by introducing plant–herbivore interactions into the existing species competition model. For simplicity, we assumed that the herbivore population size is constant throughout the plant growth period. This mirrors natural conditions when herbivore life cycles outlast the plants they feed on, such as many grazing herbivores in both terrestrial and aquatic ecosystems. For example, the aquatic nymphal stage of mayflies can last for up to 2.5 years while feeding on algae, epiphytes and aquatic plants with various life spans [44]. Our study on aquatic plant–herbivore dynamics resonates with ecological principles seen in terrestrial ecosystems, where numerous mammalian herbivores (e.g. deer) often outlast plant reproductive cycles. In other systems, however, herbivores and plants may reproduce at similar rates, or even herbivores at higher rates than plants, such as aphids feeding on duckweed [45]. This might lead to oscillations between herbivores and plants (for both species), similar to the classic predator and prey scenarios ([46] and references therein). However, in such scenarios, understanding the effects of herbivory on plant coexistence requires solving more complex analytical models. Future studies that model population dynamics of herbivores (as did in e.g. [47]), as well as mesocosm approaches that manipulate and quantify herbivore densities (as indeed snails laid eggs in our outdoor mesocosms over the course of 9 weeks), will provide further insights into the species dynamics of multitrophic communities.

To determine interspecific competition coefficients, we used model-guided assays in which we kept the abundance of the competitor constant over time while varying the starting abundance of the focal species and measuring the focal species’ *per capita* growth (fitness) (electronic supplementary material, figure S3), assuming a linear relationship. While this approach made the experiments in the lab feasible, considering nonlinearity within our model, as proposed elsewhere [13,14,48,49], will further improve the realistic characterization of the species dynamics. To quantify nonlinearity, one will need to perform experiments with a wider range of density gradients of the competitors and herbivores. While the current model analysis, considering linearity and using bootstrapped coefficients, demonstrates an alignment between the theoretical model and experimental findings in three out of four scenarios (figure 1*c*), exploring alternative methodologies holds promise for further refining and enhancing the robustness of the results.

Despite these limitations, we experimentally validated most of the predicted outcomes of our model by using three interacting species under indoor conditions. Under indoor conditions, we found that the ratio of herbivory effects on duckweed and algae is less than the ratio of interspecific competition caused by algae and intraspecific competition (figure 1, $\beta_{1}/\beta_{2}<\;a_{12}/\;a_{22}$). This predicts that snails could benefit duckweed fitness. Indeed, our results showed that snails benefitted duckweed fitness in the presence of low (0.9 [10^6^ cells ml^−1^]) and intermediate– high (4.1 [10^6^ cells ml^−1^]) starting concentrations of the competing algae. The only exception was that when competing against high (5.8 [10^6^ cells ml^−1^]) algae concentrations (figure 1*c*), the duckweed did not benefit from snail herbivory. One possible explanation is that the snail changed its feeding preference when duckweed grew together with high concentrations of algae, which might lead to an increase in the $\beta_{1}/\beta_{2}$ ratio. However, future studies are needed to test this hypothesis. As predicted by the model, algae did not benefit from the presence of duckweed under herbivory in the tested scenarios (electronic supplementary material, figure S4). In nature, snails feed on both micro- and macroalgae, which have also been found in outdoor communities [50–53]. It is likely that snails prefer algae over duckweed, as macroalgae have thinner and more easily palatable cell walls compared with macrophytes [50].

Under outdoor conditions, the algal community changed over time, which also likely led to changes in intra- and interspecific competition between algae and duckweed. Therefore, it is technically challenging to quantify model parameters directly *in situ*. Nevertheless, we found evidence that herbivory can indeed increase duckweed fitness by reducing algal abundance outdoors, which is likely due to the combined effects of the relative changes and absolute reduction of both microalgae and macroalgae. Duckweed can suppress algae growth by reducing light availability [24] and nutrients [25], while in turn, algae can produce toxins and increase the pH to levels toxic to duckweed [24,54]. Grazing snails, such as *Radix labiata*, have been observed to reduce the biomass of epiphytic algae [55]. This reduction in algae biomass leads to higher nutrient levels, subsequently promoting the growth of floating *Lemna gibba* while potentially inhibiting the growth of submerged *Ceratophyllum demersum* [55]. Moreover, the impact of free-floating plants on submerged plant growth varied based on plant density and species identity, with dense duckweed (*Lemna)* cover having a more negative effect on the growth of the submerged plant *Elodea* compared with *Ceratophyllum* [23]. Increasing nitrogen concentration led to higher *Lemna* biomass, reducing underwater light availability and algal biomass, particularly benefiting *Ceratophyllum* growth owing to indirect facilitation through reduced algal competition [23].

In addition to the aquatic ecosystems, studies have also shown that herbivory can increase plant fitness in terrestrial environments by reducing competition. For example, introducing novel herbivores to a plant community enhanced the growth of small-stature plants by reducing the dominant plant species and increasing light availability [56]. Moreover, herbivory plays a crucial role in maintaining plant diversity in grassland ecosystems by influencing competition for light, as demonstrated by the mitigation of biodiversity loss through the restoration of light availability in the understorey in the presence of herbivores [57]. Overall, these studies and our results highlight the importance of considering indirect effects, such as competition, when studying plant–herbivore interactions. From an evolutionary perspective, herbivores can not only drive the evolutionary changes in traits involved in defences but also shape the evolution of traits that are associated with intraspecific and interspecific competition in nature.

Our quantitative model describing plant–herbivore interactions in the presence of competition can be used to understand community dynamics caused by species invasions [20,58–60], which are often mediated by either invasive herbivores or fast-growing exotic plants. Current studies often focus on quantifying the effects of herbivory (either native or exotic) on plant communities (either native or exotic). However, based on our model, the trajectory of the focal plant under herbivory is not only dependent on the relative differences of herbivory on the focal plant and its competitors, but also dependent on relative differences between interspecific and intraspecific competition, a key parameter that was not quantified by most of the studies. We believe that adopting the quantitative theoretical model and quantifying these two key parameters might explain inconsistent outcomes of community responses to invasion from different studies [61,62] and offer more prediction powers for estimating community responses to species invasions.

Together, we demonstrate that herbivores determine plant fitness through both direct negative effects via feeding and indirect beneficial effects by reducing the growth of competitors. Consequently, herbivores can increase plant fitness by reducing the abundance of the competing species. Our model allows the quantification of these effects, showing that plant fitness depends on the relative ratio of intraspecific and interspecific competition, as well as the relative consumption rates of focal and competing species, respectively. Our study highlights that plant–herbivore interactions and their evolution should be studied in a community context, as indirect effects can sometimes overwrite direct effects.

## Data Availability

The datasets supporting this article have been uploaded as part of the online supplementary material. Raw sequencing data are available on NCBI SRA under BioProject PRJNA914505 (16S duckweed samples), PRJNA914509 (18S duckweeds samples), PRJNA996071 (18S algae samples). The code to replicate sequencing data analyses, as well as raw data and code for our modelling approach are available at [63] and [64]. Supplementary material is available online [65].
